# Super-Dispersed Fe–N Sites Embedded into Porous Graphitic Carbon for ORR: Size, Composition and Activity Control

**DOI:** 10.3390/nano11082106

**Published:** 2021-08-19

**Authors:** Xin Yu Wang, Ze Wei Lin, Yan Qing Jiao, Jian Cong Liu, Rui Hong Wang

**Affiliations:** 1 Key Laboratory of Functional Inorganic Material Chemistry, Ministry of Education, School of Chemistry and Material Science, Heilongjiang University, Harbin 150080, China; wangxinyu11222021@163.com (X.Y.W.); jiaoyanqing@hlju.edu.cn (Y.Q.J.); 2 School of Chemical Engineering and Chemistry, Harbin Institute of Technology, Harbin 150001, China; lzw305145514@163.com

**Keywords:** Fe-N sites, ZIF-8, super-dispersion, ORR, high catalytic performance

## Abstract

Searching for high-efficient, good long-term stability, and low-cost electrocatalysts toward oxygen reduction reaction (ORR) is highly desirable for the development of sustainable energy conversion devices. Iron–nitrogen doped carbon (Fe–N/C) catalysts have been recognized as the most promising candidates for traditional Pt-based catalysts that benefit from their high activity, excellent anti-poisoning ability, and inexpensiveness. Here, a super-dispersed and high-performance Fe–N/C catalyst was derived from chemically Fe-doped zeolitic imidazolate frameworks (ZIFs) by directly bonding Fe ions to imidazolate ligands within 3D frameworks. It produced a series of catalysts, whose sizes could be tuned in the range from 62 to over 473 nm in diameter. After rationally regulating the component and heating treatment, the best ORR activity was measured for the catalyst with a size of 105 nm, which was obtained when the Fe^3+^/Zn^2+^ molar ratio was 0.05 and carbonization temperature was 900 °C. It exhibited a high onset potential (E_onset_ = 0.99 V) and half-wave potential (E_1/2_ = 0.885 V) compared with a commercial 20% Pt/C catalyst (E_onset_ = 0.10 V, E_1/2_ = 0.861 V) as well as much better durability and methanol resistance in an alkaline electrolyte.

## 1. Introduction

The oxygen reduction reaction (ORR) is one of the crucial electrochemical processes for clean energy devices such as fuel cells [1,2] and metal–air batteries [3,4,5]. However, a large amount of Pt is required to catalyze the kinetically sluggish ORR at the cathode, which therefore severely restricts their widespread commercialization [6,7]. Employment of high-performance, non-precious metal ORR catalysts is considered as a long-term goal for the evolution of these technologies.

Recently, the coordinately unsaturated transition metal–nitrogen sites confined within the carbon matrix have been widely explored as efficient and promising non-precious metal catalysts that many hope may be able to replace Pt-based catalysts toward ORR [8,9,10,11]. To date, Fe–N/C catalysts are one of the most brilliant candidates due to their excellent ORR activity, stability, and instinctive anti-poisoning power [12,13,14,15,16,17]. Combining the theoretical calculations with the experimental results, the co-doping of Fe and N in the form of iron–nitrogen (Fe–N) is considered to provide active sites for O_2_ adsorption and the subsequent breaking of O=O bonding in ORR [18,19]. Hence, many efforts have been devoted to increasing the Fe–N site with the carbon matrix, which have led to encouraging performance improvement. However, undesirable aggregation of metal atoms is prone to forming during high-temperature pyrolysis with increasing metal content, which leads to a decline in catalytic activity [20,21]. Therefore, it is highly desirable to explore the super-dispersed, especially the atomically-dispersed Fe–N sites, at a high density embedded into a favorable carbon matrix.

Metal-organic frameworks (MOFs), constructed from central metal ions and ligands, have attracted tremendous attention as precursors for the synthesis of non-precious metal catalysts [22,23,24,25,26,27]. MOF-derived catalyst materials can not only inherit their parents’ morphology, but also contain all the necessary elements (metal, carbon, and nitrogen) as well as tunable chemical composition. More recently, the zinc-based zeolite imidazole framework (ZIF-8) has been widely studied to encapsulate organic [28,29] or inorganic [30,31,32] iron-containing molecules, which were then subjected to pyrolysis at high temperature under an inert gas to prepare the Fe–N–C catalysts. Due to the uniform dispersion of Fe species in precursors, the obtained catalyst possesses the high-dispersed Fe–N sites confined within the carbon matrix. Although these studies have made significant achievements in ORR electrocatalytic activity, there is still room for improvement. Meanwhile, the particle size and distribution play an important role in the ORR performance; however, the detailed investigation of the structure–performance correlations is limited.

In this work, a series of Fe-doped ZIF precursors with different Fe^3+^/Zn^2+^ molar ratios were easily synthesized through a chemical doping approach. In this process, the Fe ions could partially replace Zn ions and coordinate with imidazole ligands, which enable them to be highly dispersed in the organic skeleton. After one-step pyrolysis, porous carbon with a hierarchical structure was obtained, in which the Fe in the form of Fe–N were super-dispersed at an atomic scale. The size of the catalyst is tunable over a wide range from 62 to 473 nm, which can be tailored by rationally regulating the component and heating treatment. More attention was paid to the structure–performance correlations; as a result, the best optimized ORR activity was achieved on a catalyst of 105 nm, which exhibited a half-wave potential of 0.885 V, limit current of 4.8 mA cm^-2^, and better long-time durability than the commercial 20% Pt/C catalyst. 

## 2. Materials and Methods

### 2.1. Synthesis of Fe-ZIF Precursors

A wide range of crystal sizes of Fe-doped ZIF was controlled by tuning the molar ratios of Fe(NO_3_)_3_·9H_2_O and Zn(NO_3_)_2_·6H_2_O to 0.025:1, 0.05:1, and 0.075:1. Typically, in the case of a Fe^3+^/Zn^2+^ molar ratio of 0.05:1, 1 mmol Zn(NO_3_)_2_·6H_2_O (297 mg) and 0.05 mmol Fe(NO_3_)_3_·9H_2_O (20 mg) are dissolved in 40 mL methanol and stirred to form a clear solution. This solution was subsequently poured into another clear solution containing 328 mg of 2-methylimidazole dissolved in 40 mL of methanol. Then, the mixed solution was heated at 100 °C for 12 h. After cooling to room temperature, the product was collected via centrifugation and washed with methanol several times. After drying at 60 °C under a vacuum overnight, a precursor named Fe–ZIF-0.05 was obtained. Similarly, the Fe–ZIF precursors with Fe^3^^+^/Zn^2+^ molar ratios of 0.025:1 and 0.075:1 were also synthesized based on the same process and named as Fe-ZIF-0.025 and Fe-ZIF-0.075, respectively. 

### 2.2. Synthesis of Fe-N/C Catalyst

First, Fe–ZIF precursors with different Fe^3+^/Zn^2+^ molar ratios were carbonized at 900 °C in N_2_ with a heating rate of 5 °C min^−1^ to achieve pyrolysis and graphitization. After cooling to room temperature, the black powder was put into 100 mL 0.5 M H_2_SO_4_ solution and heated at 80 °C for 10 h to etch the unstable inorganic metal phase. In the end, the final catalysts of Fe–N/C (Fe–N/C–0.025–900), Fe–N/C–0.05–900, and Fe–N/C–0.075–900) were obtained after centrifugation and separation. In order to explore the effect of heat treatment temperature on the morphology and activity, the Fe–ZIF–0.05 precursor was processed at different temperatures (600–1000 °C) by the same method, and the products were denoted as Fe–N/C-0.05-600, Fe–N/C-0.05-700, Fe–N/C-0.05-800, Fe–N/C-0.05-900, and Fe–N/C-0.05-1000, respectively. 

### 2.3. Material Characterization

X-ray diffraction (XRD) patterns were performed with an X-ray diffractometer (XRD, Bruker D8 Advanced) with an accelerating voltage of 40 kV. The Raman data were collected using a Raman spectrometer (Jobin Yvon HR800). The morphologies of the synthesized samples were studied by SEM (Philips XL-30-ESEM-FEG, 5–20 kV). The Brunauer–Emmett–Teller (BET) surface area of the product was measured by using N_2_ adsorption/desorption (TriStar II 3020). Transmission electron microscopy (TEM), high-resolution TEM (HRTEM), dark-field scanning transmission electron microscopy (STEM), and energy-dispersive X-ray measurement (EDX) were implemented on TEM JEOL JEM-3010. The chemical state and surface composition were investigated by the X-ray photoelectron spectroscopy (XPS, VG ESCALAB MK II). The surface area and average pore width were measured on a Micromeritics TrisStar II 3020. 

### 2.4. Electrochemical Measurements

Electrochemical properties were tested using a Pine electrochemical workstation by a standard three-electrode system at room temperature, in which a rotating ring-disk electrode (RRDE, area = 0.2470 cm^2^) was used as the working electrode and a Pt flake and reversible hydrogen electrode (RHE) were adopted as the counter and reference electrodes, respectively. The catalyst ink was prepared by ultrasonically dispersing a mixture containing 5 mg of catalyst, 0.8 mL of isopropyl alcohol, 0.2 mL of deionized water, and 30 μL of Nafion (5 wt%). Subsequently, 30 mL of the catalyst ink was pipetted onto a pre-cleaned working electrode. The ORR performance was recorded at 1600 rpm with a scan rate of 5 mV s^−1^ in O_2_-saturated 0.1 M KOH at room temperature, and the potentials are given versus a reversible hydrogen electrode (vs. RHE). The loading amount on RRDE was 0.589 mg cm^−2^, and the potential range was controlled in the range of 0–1.2 V for all measurements. Chronoamperometry (CA) was performed to assess the durability and methanol tolerance for catalysts under 0.885 V for 10,000 s with a rotating speed of 1600 rpm.

## 3. Results

### 3.1. Structural Characterization of Fe-N/C Catalyst

**Figure 1:**
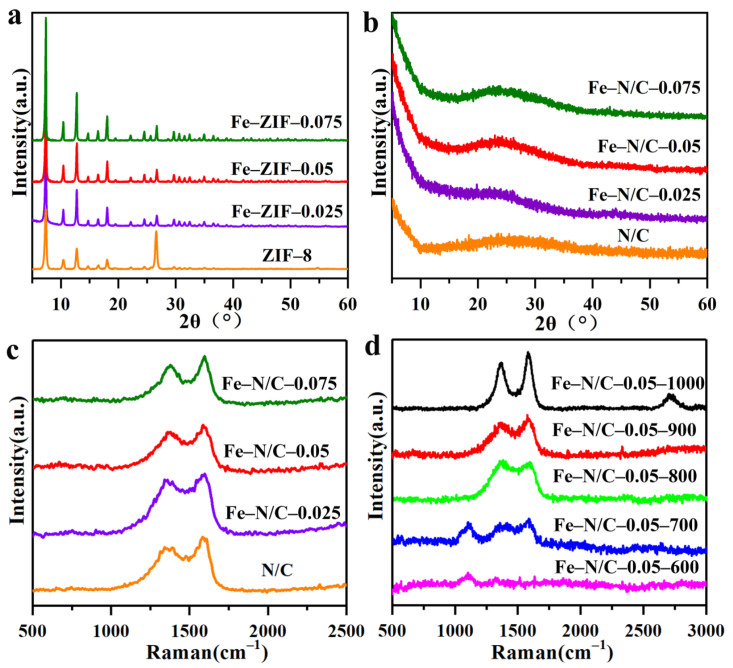
(**a**) The XRD patterns of the precursors with different Fe feeding, (**b**) XRD patterns, (**c**) Raman spectra of products synthesized under 900 °C, and (**d**) Raman spectra of products by carbonizing Fe–ZIF–0.05 precursors at different temperatures.

Figure 1a displays the XRD patterns of the pure ZIF-8 and Fe-ZIF precursors with different Fe^3+^/Zn^2+^ molar ratios. All of the samples exhibited the characteristic peaks at 7.38°, 10.42°, 12.77°, 14.75°, 16.50°, and 18.08°, which are ascribable to the (101), (002), (112), (200), (013), and (222) planes of ZIF-8, respectively [33]. These results demonstrate that the Fe–ZIF precursors inherited the original structure from pure ZIF-8, and that the introduction of low-concentration Fe^3+^ did not change the structure of the ZIF-8 framework. Despite no shift in the diffraction peak being observable with the increasing molar ratios of Fe^3+^/Zn^2+^, the peak intensity was significantly enhanced, indicating the improved crystallinity of Fe–ZIF precursors. Subsequently, all the precursors including the pure ZIF-8 were carbonized at 900 ℃ in a N_2_ atmosphere. The XRD patterns of products after carbonation are exhibited in Figure 1b, where the wide peaks between 2θ of 20 and 30° are typical characteristics of carbon material derived from ZIF-8 [34]. 

Raman spectroscopy was adopted to further reveal the crystallinity of carbon. As displayed in Figure 1c, two distinct peaks at 1588.24 and 1359.91 cm^−1^ can be ascribable to the G-band and D-band of graphite, respectively, corresponding to the sp^2^ bonds of graphitic structure and the sp^3^ bonds of defects in the carbon matrix [35,36]. The I_G_/I_D_ value represents the ratio of the degree of defect density and the degree of graphitization of materials [36,37]. For the pure carbon derived from ZIF-8, the intensity ratio of G-band to D-band (I_G_/I_D_) was 1.168, which proves that the product was graphited due to the catalytic effect of the transition metal at a high temperature. For the carbon materials obtained from Fe–ZIF precursors, the I_G_/I_D_ values were 1.184, 1.162 and 1.159, indicating that the graphitization of carbon was improved with increased iron content. 

To further investigate the effect of carbonization temperatures on the degree of graphitization (Figure 1d), the Fe–ZIF–0.05 precursor was treated at different temperatures (600–1000 °C). At a temperature of 600 °C, the Raman band at 1115 cm^−1^ is a typical characteristic spectra for Fe–zeolite imidazole skeleton [38]. When the temperature was up to 700 °C, the Raman peaks of G-band and D-band appeared, respectively. As the temperature increased to 800 °C, the Raman peak corresponding to Fe–ZIF8 disappeared, indicating the complete disintegration of the imidazole skeleton at 800 °C and the I_G_/I_D_ value was calculated to be 1.159. Upon further increasing the temperature to 900 °C, the I_G_/I_D_ value was calculated as 1.162, corresponding to a typical graphitic carbon [39]. The graphitization was further improved with the increased temperature, and an obvious 2D peak was found at 1000 °C. These results show that a high temperature is beneficial to the formation of graphitization. 

**Figure 2:**
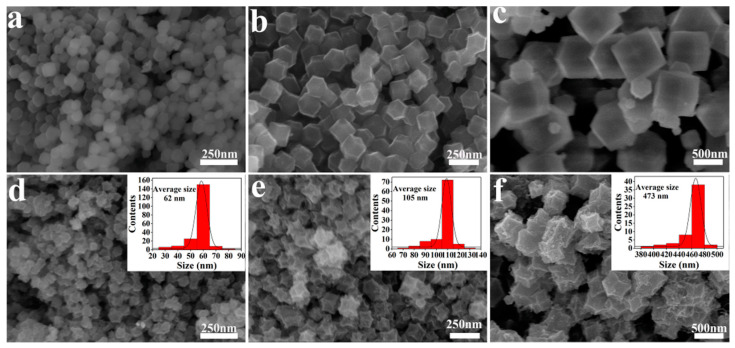
The SEM images of precursors (**a**) Fe–ZIF–0.025, (**b**) Fe–ZIF–0.05, (**c**) Fe–ZIF–0.075 and their carbonized products at 900 °C, (**d**) Fe–N/C–0.025–900, (**e**) Fe–N/C–0.05–900, and (**f**) Fe–N/C–0.075–900.

Next, the morphology and structural characteristics of Fe–ZIF precursors with different Fe^3+^/Zn^2+^ molar ratios and their products after carbonation at 900 °C were investigated through SEM. As shown in Figure 2a–c, all the Fe–ZIF precursors displayed the typical dodecahedron structure of ZIF-8, and evenly in a uniform distribution without significant aggregation. The size of precursors was tunable over a wide range by changing the concentration of metal salts and ligands. For the Fe–ZIF–0.025 sample, the precursor size was 90 nm, which increased with the increase in iron content and reached 200 and 900 nm, respectively, for Fe–ZIF–0.05 and Fe–ZIF–0.075. Figure 2d–f exhibits the SEM images of the carbonized products at 900 °C. It is evident that the products have inherited the main morphology of Fe–ZIF precursors. Due to the dehydration and pyrolysis of organic ligands, the dodecahedral structure collapsed toward the center, resulting in a gradual decrease in size. The average size of products is 62, 105, and 473 nm for Fe–N/C–0.025–900, Fe–N/C–0.05–900, and Fe–N/C–0.075–900, respectively. Moreover, the Zn atoms in the ZIF structure was easily evaporated (>907 °C), resulting in the porous carbon structures in the products [40]. As can be observed directly from Figure 2d–f, the surface roughness was improved with the increase in Fe^3+^/Zn^2+^ molar ratios. 

**Figure 3:**
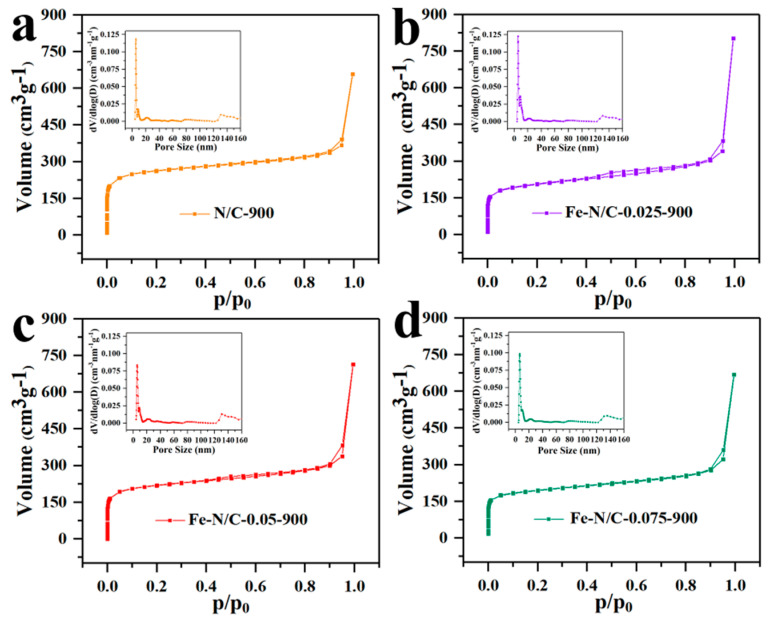
N_2_ adsorption and desorption isotherms and pore-size distributions of Fe–N/C-900 with different moral ration of iron.

N_2_ adsorption/desorption isotherms were measured to evaluate the trends of porosity change with different Fe^3+^/Zn^2+^ molar ratios. As presented in Figure 3, the nitrogen uptake for all samples exhibited the typical Type-I isotherm at the low relative pressure, demonstrating the existence of a microporous structure. At higher relative pressure, a significant hysteresis of the adsorption/desorption process is clearly noticeable, which is indicative of the Type-IV isotherm for mesoporous materials [30]. The porous structure partly originates from the abundant micropores in ZIF-8; moreover, the subsequent vapor of metal Zn above its boiling point (>907 °C) would also leave it porous in the carbon skeleton. Table 1 gives the detailed trend of the Brunauer–Emmett–Teller (BET) surface area and total pore volume change with the increasing Fe^3+^/Zn^2+^ molar ratios. It was found that the total pore volume gradually decreased with increasing iron concentration. This phenomenon is due to the increase in iron content, which would reduce the inter-particle space available and lower their surface-to-volume ratio. On the other hand, graphitization and specific surface area are contradictory. In other words, the higher the iron content, the more conductivity to improve the electron transfer in ORR; however, this comes with the loss of the specific surface area. Therefore, the surface area, pore structure, and graphitization degree of the product can be controlled by adjusting the iron content. As summarized in Table 1, Fe–N/C–0.05–900 displayed the surface area and total pore volume of 812.1 m^2^ g^-1^ and 1.105 cm^3^ g^-1^, respectively, which was medium among all the samples. Such a large specific surface area structure can greatly increase the number of exposed active sites, and the hierarchical micro/meso-porous structure would simultaneously boost the electrochemical activation and mass-transport properties. Hence, we elucidated that the Fe–N/C–0.05–900 sample would be a good potential ORR electrocatalyst. The effect of heating treatment temperature on product size was also investigated on the Fe–N/C–0.05 precursor (Figure S1). The average size was 181, 159, 136, 105 and 90 nm, for 600, 700, 800, 900 and 1000 °C, respectively. Therefore, it can be concluded that rising temperature causes the dodecahedron structure to collapse gradually. The influence of catalyst size on ORR activity is also discussed in detail in the performance section. 

**Figure 4:**
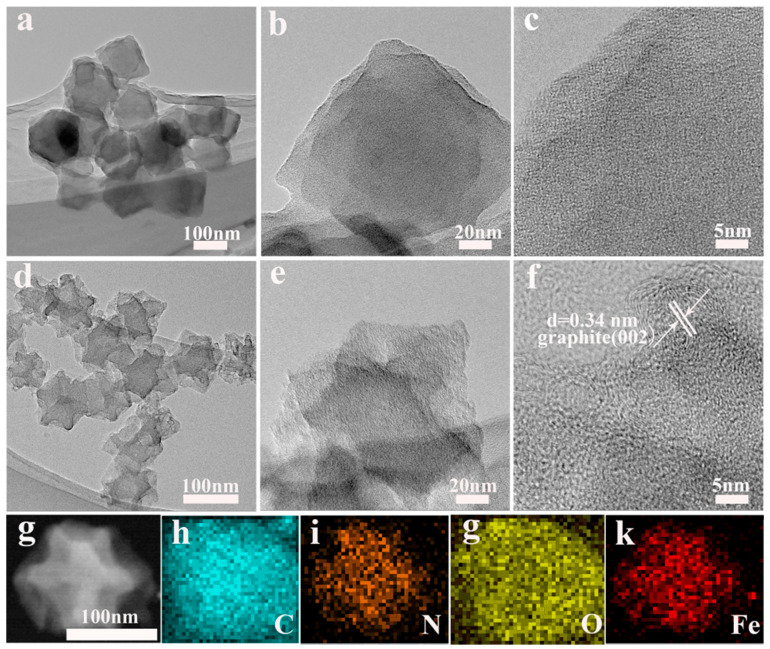
TEM images of the precursors Fe–ZIF–0.05 (**a**–**c**), Fe–N/C–0.05–900 (**d**–**f**), HAADF-STEM images and elemental mapping of Fe–N/C–0.05–900 (**g**–**k**).

The microscopic structure of the Fe–ZIF–0.05 precursor and the Fe–N/C-0.05–900 sample was investigated by using TEM and HRTEM. As exhibited in Figure 4a–c, the precursor showed a typical dodecahedral structure with a smooth surface. The particle size was approximately 200 nm, which is consistent with the result of the SEM results. After being carbonized at 900 °C, the dodecahedron structure shrank, with the surface becoming rougher than before (Figure 4d–f). Interestingly, no metal particles or aggregates were detected in the product, which proves that the Fe species should be uniformly dispersed in the whole carbon framework at the atomic level, thereby providing a large number of active sites for the electrocatalytic reaction. Notably, a large number of porous structures were detected on the surface of the sample, which are thought to have resulted from the volatilization of Zn atoms during the carbonization process. 

To further observe the microstructures, the high-resolution TEM (HRTEM) was performed. Figure 4f exhibits the visible fringes with an interplanar spacing of 0.34 nm, which corresponds to the (002) crystal plane of graphite carbon, further proving the formation of graphitization after the carbonization at high temperature. The formation of graphitic carbon is contributed from the catalytic effect of Fe species. This result is also consistent with those of the Raman spectra. Due to the limitations of our experimental conditions, we could not carry out spherical aberration-corrected transmission electron microscope (SACTEM) or the X-ray adsorption fine structure (XAFS) to directly identify the atomic Fe sites embedded into carbon. However, we have attempted to improve the molar ratio of Fe^3+^/Zn^2+^ to 0.15 in order to investigate the composition, structure, and morphology of products with increased iron content. The XRD pattern in Figure S2a indicates that Fe–ZIF–0.15 precursor still inherited the original structure from pure ZIF-8, which is consistent with the other proportional precursor. However, the obvious XDR diffraction peaks for graphitized carbon at 2θ of 26.2° and the identifiable lattice fringe with d = 0.34 nm were both detected. These results can be attributed to the Fe specified, which are beneficial to the formation of graphitic carbon at a high temperature. More interestingly, the characteristic diffraction peak for iron at 2θ = 45.9° was simultaneously observed, which had a good fit with the equally distributed iron nanoparticles in Figure S2c,d. This result demonstrates that increasing the iron content will result in the aggregation of atomic iron. In turn, in the case of a very low content of iron, it is super-distributed in the skeleton at the atomic level. Figure 4g–k shows the dark-field scanning transmission electron microscopy (STEM) and EDX elemental mapping of Fe–N/C–0.05–900. All the atoms comprising Fe, N, C, and O were evenly distributed in the carbon nano skeleton. More importantly, it provides evidence to confirm the existence of iron atoms. The sum spectrum of the maps is also displayed in Figure S3. 

**Figure 5:**
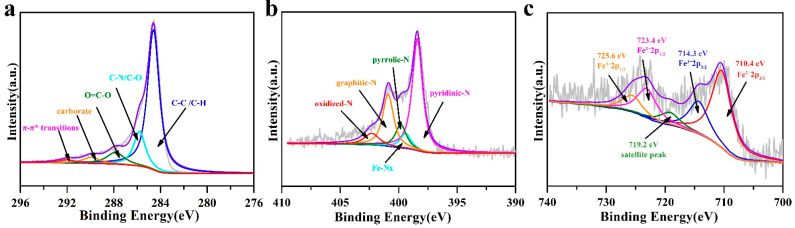
XPS spectra of Fe–N/C–0.05–900: (**a**) C 1s, (**b**) N 1s, and (**c**) Fe 2p.

X-ray photoelectron spectroscopy (XPS) was applied to probe the surface elemental and bonding configurations of Fe–N/C–0.05–900. Figure S4 shows the full spectrum of the Fe–N/C–0.05–900 sample. Although the intensity of the carbon signal was very high, a fine scan for Fe was still detectable. The element atomic contents of Fe, C, N, and O in Fe–N/C–0.05–900 were measured to be 1.20, 87.76, 7.34 and 3.70 at%. (Table S1). As for the C 1s (Figure 5a), the peaks at 284.6 eV, 285.8 eV, 287.8 eV, 289.9 eV and 292.0 eV were due to the C–C/C–H, C–N/C–O, O=C–O, carbonate, and π–π* bonds, respectively. These results confirm the formation of N-doped carbon [41] and graphitized carbon [42]. Focusing on the high-resolution XPS spectrum of N 1s (Figure 5b), the peaks at 398.2 eV, 399.8 eV, 400.8 eV and 402.7 eV can be attributed to the coordination environments of pyridinic–N [43], pyrrolic–N [44], graphitic–N [45], and oxidized–N [46]. These findings indicate that N elements have been successfully incorporated into the carbon matrix, in which pyridinic N as reported would act as the active component to modify the electronic structure of carbon and coordinate with the metal forming the highly active Fe–Nx moieties [20,47], while graphitic N could show a synergistic effect in helping O–O bond splitting [19]. Accordingly, the XPS peak of N 1s at bonding energy of 399.0 eV was identified to be Fe–Nx, which acts as the active sites for the ORR [48]. Furthermore, the presence of Fe in the Fe–N/C–0.05–900 can also be validated by the evidence that several Fe peaks including Fe (II) 2p3/2 (710.4 eV), Fe (III) 2p3/2 (714.3 eV), Fe (II) 2p1/2 (723.4 eV), and Fe (III) 2p1/2 (725.6 eV) can be observed in Figure 5e. In addition, the satellite peak was observed at 719.2 eV.

### 3.2. ORR Catalytic Results

**Figure 6:**
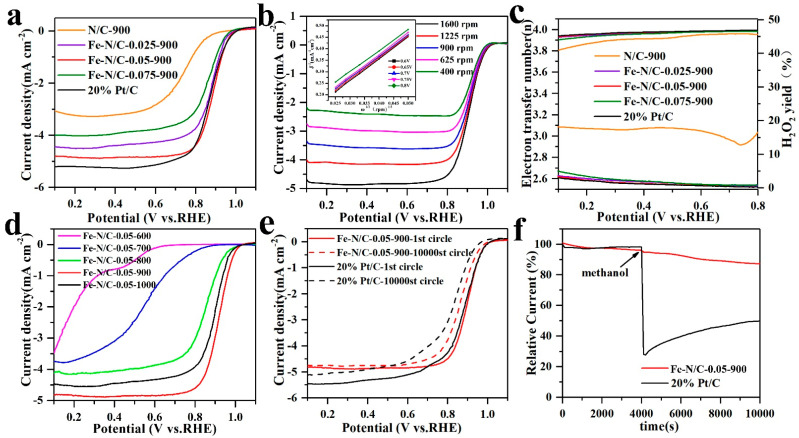
(**a**) LSV curves of Fe–N/C with different molar ratios of iron and 20% Pt/C in O_2_–saturated 0.1 M KOH, (**b**) LSV curves of Fe–N/C–0.05–900 obtained at different rotation rates and K–L plots, (**c**) transferred electron numbers and H_2_O_2_ yield at different potentials, (**d**) ORR curves of the samples at different temperatures in 0.1 M KOH, (**e**) Stability tests of Fe–N/C-0.05-900 and 20% Pt/C catalysts: the LSV curves before and after 10,000 times CV. (**f**) Chronoamperometric responses of Fe–N/C-0.05-900 and 20% Pt/C, in which 3 M methanol was added at 4000 s.

The electrocatalytic activity of the Fe–N/C samples with different Fe^3+^/Zn^2+^ molar ratios were evaluated in a three-electrode system. As shown in Figure 6a, the ORR polarization curves were recorded in oxygen saturated 0.1 M KOH, and invested the influence of Fe content on electrocatalytic performance. Obviously, all the catalysts embedded with a small amount of Fe were more active in terms of ORR activity than the pure ZIF-8 (N/C) in alkaline medium. Among all the Fe-containing samples, the Fe–N/C-0.05-900 sample displayed the best catalytic activity with a high onset potential (E_onset_ = 0.99 V), which is comparable to a commercial 20% Pt/C (E_onset_ = 0.10 V) and many reported Fe-N/C-based catalysts (Table S2). Furthermore, the half-wave potential of the Fe–N/C–0.05–900 sample (E_1/2_ = 0.885 V) shifted forward by approximately 24 mV than 20% Pt/C catalyst (E_1/2_ = 0.861 V), indicating that the Fe–N/C–0.05–900 catalyst had similar ORR reaction kinetics to standard Pt/C catalysts, and is more prominent in the region of kinetics and diffusion mixing control. 

The electron transfer kinetics of Fe–N/C–0.05–900 was further investigated by LSV curves at different rotation speeds (400–1600 rpm). As displayed in Figure 6b, the current densities of Fe–N/C–0.05–900 increased with the increase in rotation speed, suggesting the shortened diffusion distance of oxygen at high rotating speeds. The inset in Figure 6b illustrates the good linearity of K–L plots for Fe–N/C–0.05–900, reflecting the first order reaction kinetics related to the O_2_ concentration. In addition, based on the K–L equation, the electron transfer numbers (n) of Fe–N/C–0.05–900 (*n* = 3.91) and 20% Pt/C (*n* = 3.95) were determined to be close to 4, indicating that the catalyst follows a dominant four-electron pathway when performing ORR electrocatalysis (Figure 6c). The H_2_O_2_ yield of all the Fe–N/C catalysts was less than 5%, revealing that these materials toward ORR catalysis are mainly based on the 4e^-^ reaction pathway. Figure S5 shows the Tafel plots derived from the kinetic-controlled region in the ORR polarization curves. Compared with the well-established 20% Pt/C, the Fe–N/C–0.05–900 catalyst exhibited an approximate Tafel slope, further indicating their similar ORR mechanisms and kinetic characteristics to 20% Pt/C [37].

The various versions of Fe–N/C–0.05 catalysts obtained by carbonizing the Fe–ZIF-0.05 precursor at 600, 700, 800, 900 and 1000 °C were extensively investigated, respectively. The LSV voltammograms in O_2_-saturated 0.1 M KOH solution at a rotating speed of 1600 rpm are compared in Figure 6d. It was found that the measurable steady-state ORR current could be detected for both the Fe–N/C–0.05–600 and Fe–N/C–0.05–700 samples, although the onset potential and half-wave potential were very low. When the temperature was further increased to 800 °C, the ORR activity exhibited a significant improvement. To understand this appearance, the overall N 1s and C 1s determined by XPS are summarized in Figure S6. The N elemental quantification analysis (Table S3) indicates that increasing the heating temperature led to a decline in the total N content. Nevertheless, the continuous decrease in the N content did not lead to a decline in activity. This result is coincident with the reported reference [20], demonstrating that the N content in the catalysts might be sufficient. It is worth noting that a new bond of Fe–Nx at 399.0 eV formed at 800 °C (Figure S6a), which suggests that 800 °C is the crucial point for generating active sites toward ORR. The maximum activity was reached at 900 °C. This can be attributed to (i) the formation of a larger number of active sites; (ii) graphitization increasing with temperature by the gradual narrowing of C−C peaks in (Figure S6b); and (iii) the medium particle size (105 nm), proper total pore volume (1.105 cm^3^ g^−1^), and high surface area (812.1 m^2^ g^−1^). All these reasons affect the ORR catalytic performance. When the temperature was further increased up to 1000 °C, it was found that although the *E*_onset_ and *E*_half-wave_ did not change significantly, the *J*_lim__it_ decreased obviously. We attribute this result to the collapse of the product structure at a high temperature, which will affect the diffusion of the electrolyte in the electrode material, resulting in the decay of activity.

The stability of the catalyst is an important indicator to evaluate the ORR performance. Figure 6e shows the ORR polarization curves of Fe–N/C–0.05–900 and 20% Pt/C after 10,000 CV tests in O_2_-saturated 0.1 M KOH. The half-wave potential of Fe–N/C–0.05–900 only shifted negatively by 32 mV, while the commercial 20% Pt/C catalyst shifted negatively by 57 mV, indicating the much better stability of Fe–N/C–0.05–900 compared with commercial 20% Pt/C. Furthermore, the stability of Fe–N/C–0.05–900 and 20%Pt/C was further evaluated by chronoamperometry (I/T) at a constant potential of 0.885 V in an oxygen saturated 0.1 M KOH electrolyte. As shown in Figure S7, the Fe–N/C–0.05–900 electrocatalyst maintained 90.1% of the initial current density after 10,000 s, while the current value for 20% Pt/C decreased by 14.6%, indicating the more superior durability of Fe–N/C–0.05–900 to the commercial 20%Pt/C catalyst. Such remarkable stability is thought to be due to the unique porous graphitic carbon structure derived from the ZIF-8 framework, which would act as an anchor to stabilize Fe specified in the composite and prevent Fe–N active sites from aggregation and migration in the process of electrocatalysis. Finally, with the addition of 3 M methanol, significant methanol oxidation current response was observed for Pt/C, while this was not observed for Fe–N/C–0.05–900, demonstrating its excellent resistance to methanol poisoning (Figure 6f). 

## 4. Conclusions

In summary, a series of Fe–N/C catalysts composed of super-dispersed Fe–N active sites embedded into porous graphitic carbon without metallic agglomeration were synthesized via the chemical doping of Fe ions into ZIF-8 frameworks and suffered from one-step thermal treatment in N_2_ gas. The Fe–N/C catalyst inherited the porous structure of ZIF-8 including some microporous and abundant mesoporous that generated more effective porosity for mass transfer in ORR. During the conversion, the particle size and composition of catalysts can be controlled by adjusting the Fe^3+^/Zn^2+^ molar ratio and heating temperature, which allowed us to study the size-dependent ORR activity of Pt-free catalysts. More importantly, there was no agglomeration when the content of Fe was very low. The Fe–N specified were highly-dispersed into the porous carbon phases, which exposed a large number of active sites and finally drove the more excellent ORR activity, stability, and poison tolerance, even being superior to a commercial Pt catalyst. 

## Figures and Tables

**Table 1:** BET surface areas and total pore volumes of the as-prepared samples.

	N/C–900	Fe–N/C–0.025–900	Fe–N/C–0.05–900	Fe–N/C–0.075–900
Total pore volume (cm^3^ g^−1^)	1.019	1.242	1.105	1.035
Specific surface area (m^2^ g^−1^)	983.2	754.6	812.1	725.5

## Supplementary Materials

The following are available online at https://www.mdpi.com/article/10.3390/nano11082106/s1, Figure S1: (a) SEM image of Fe–ZIF–0.05 precursor and (b-f) SEM images of Fe–N/C–0.05–600, Fe–N/C–0.05–700, Fe–N/C–0.05–800, Fe–N/C–0.05–900 and Fe–N/C–0.05–1000. The illustration shows the particle size distribution, Figure S2: (a) XRD and (b-d) TEM images of Fe–N/C–0.15–900, Figure S3: The sum spectrum of the maps for Fe–N/C–0.05–900, Figure S4: The full spectrum of Fe–N/C–0.05–900, Table S1: The element atomic contents of Fe, C, N and O in Fe–N/C–0.05–900 by XPS measurement, Table S2: The comparison of ORR activity between our work and the references, Figure S5: Tafel curves of Fe–N/C–0.05–900 and 20% Pt/C catalysts, Figure S6: The evolution of XPS N 1s (a) and C 1s (b) spectra of the Fe–N/C catalysts with increasing heating temperature up to 1000 °C, Table S3:The N elemental quantification determined by XPS analysis, Figure S7: Chronoamperometric curves of Fe–N/C–0.05–900 and 20%Pt/C electrodes in O_2_-saturated 0.1 M KOH at a rotating speed of 1600 rpm, the potential was controlled at 0.75 V for 10000 s.
