# Correction: Wang et al. Super-Dispersed Fe–N Sites Embedded into Porous Graphitic Carbon for ORR: Size, Composition and Activity Control. *Nanomaterials* 2021, *11*, 2106

**DOI:** 10.3390/nano15110854

**Published:** 2025-06-03

**Authors:** Xin Yu Wang, Ze Wei Lin, Yan Qing Jiao, Jian Cong Liu, Rui Hong Wang

**Affiliations:** 1 Key Laboratory of Functional Inorganic Material Chemistry, Ministry of Education, School of Chemistry and Material Science, Heilongjiang University, Harbin 150080, China; wangxinyu11222021@163.com (X.Y.W.); jiaoyanqing@hlju.edu.cn (Y.Q.J.); 2 School of Chemical Engineering and Chemistry, Harbin Institute of Technology, Harbin 150001, China; lzw305145514@163.com

In the original publication [1], there was a mistake in the legend for Figure 1b XRD patterns, where the XRD patterns for Fe-N/C-0.05 and Fe-N/C-0.025 overlap significantly. The correct Figure 1 legend appears below. The authors state that the scientific conclusions are unaffected. This correction was approved by the Academic Editor. The original publication has also been updated.

## Figures and Tables

**Figure 1:**
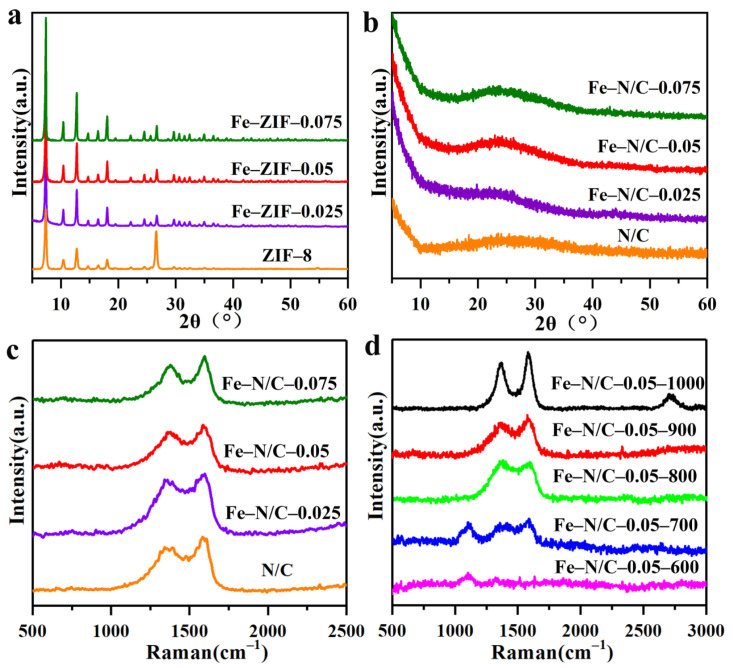
(**a**) The XRD patterns of the precursors with different Fe feeding, (**b**) XRD patterns, (**c**) Raman spectra of products synthesized under 900 °C, and (**d**) Raman spectra of products by carbonizing Fe–ZIF–0.05 precursors at different temperatures.
