# Supplementary material for: Super-Dispersed Fe–N Sites Embedded into Porous Graphitic Carbon for ORR: Size, Composition and Activity Control
Source: Nanomaterials (Basel). 2021 Aug 19;11(8):2106. doi: 10.3390/nano11082106 (PMC8399882; doi:10.3390/nano11082106)
Supplement: Supplementary file 1 [file nanomaterials-11-02106-s001.zip › nanomaterials-1307402-supplementary.pdf]

nanomaterials

Supporting Information

Super-Dispersed Fe–N Sites Embedded into Porous Graphitic Carbon for ORR: Size, Composition, and Activity Control

Xin Yu Wang [1]; Ze Wei Lin [2]; Yan Qing Jiao [1]; Jian Cong Liu [1,*]; Rui Hong Wang [1,*]

[1] Key Laboratory of Functional Inorganic Material Chemistry, Ministry of Education, School of Chemistry and Material Science, Heilongjiang University, Harbin 150080, China; wangxinyu11222021@163.com (X.W.); jiaoyanqing@hlju.edu.cn (Y.J.)

[2] School of Chemical Engineering and Chemistry, Harbin Institute of Technology, Harbin 150001, China; lzw305145514@163.com

* Correspondence: liujiancong@hlju.edu.cn (J.L.); wangruihong@hlju.edu.cn (R.W.); Tel.: +86-0451-86604331

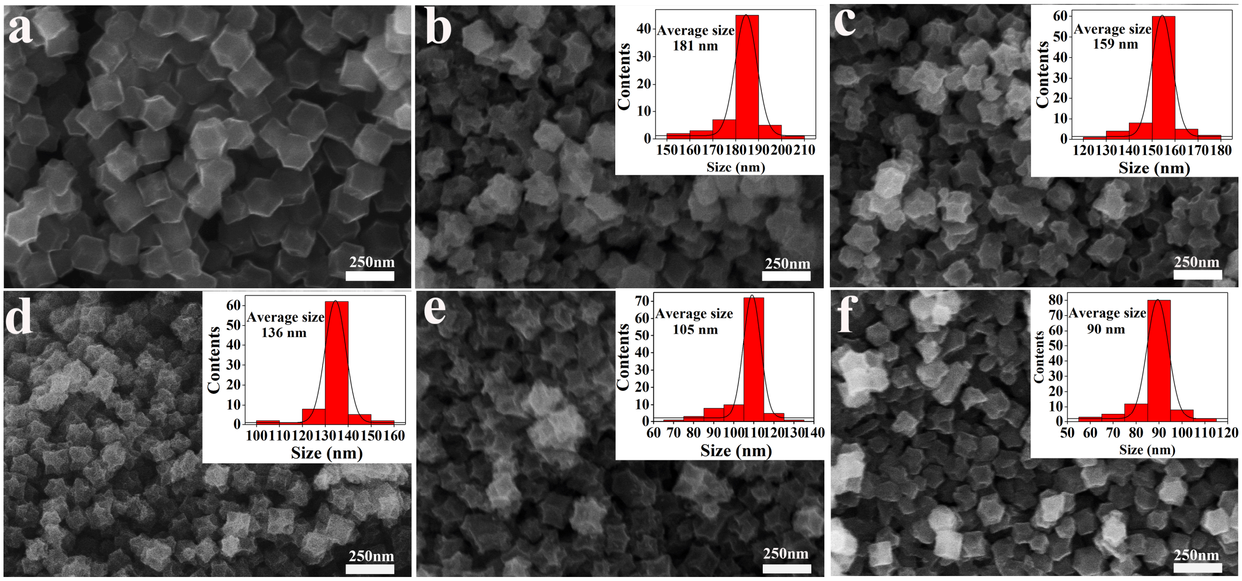

Figure S1. (**a**) SEM image of Fe-ZIF-0.05 precursor and (**b–f**) SEM images of Fe-N/C-0.05-600, Fe-N/C-0.05-700, Fe-N/C-0.05-800, Fe-N/C-0.05-900 and Fe-N/C-0.05-1000. The illustration shows the particle size distribution.

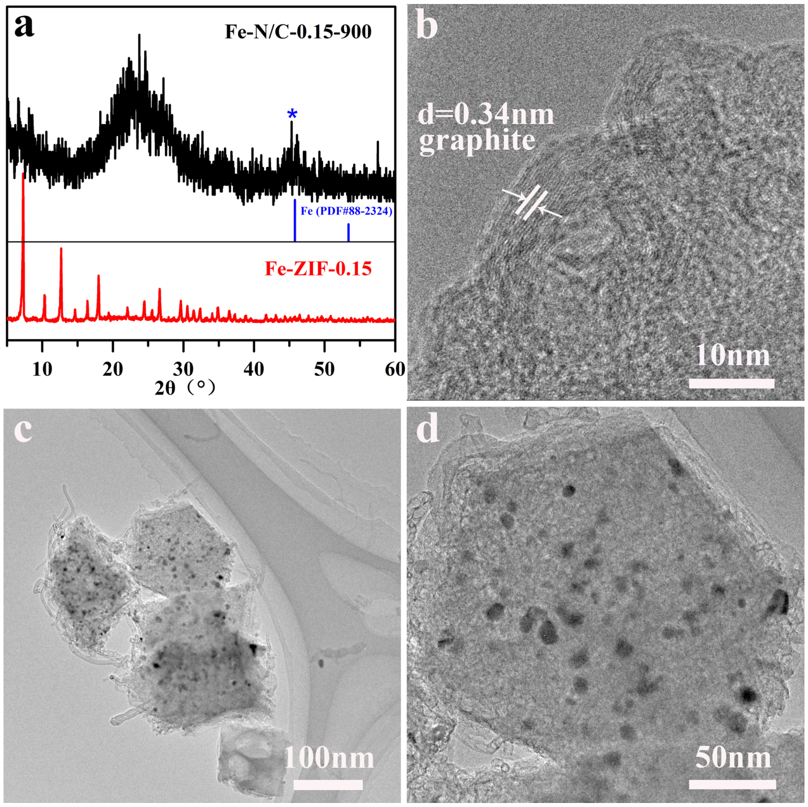

Figure S2. (**a**) XRD and (**b–d**) TEM images of Fe-N/C-0.15-900

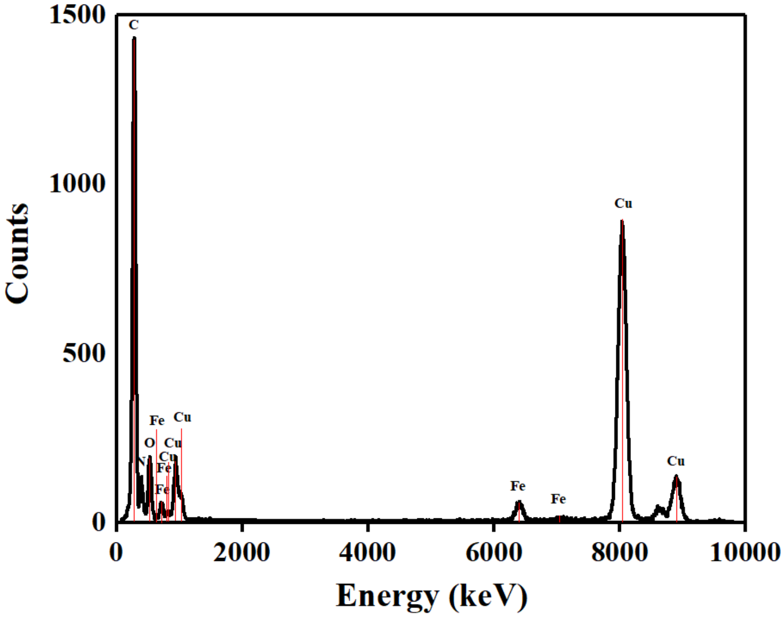

Figure S3. The sum spectrum of the maps for Fe-N/C-0.05-900

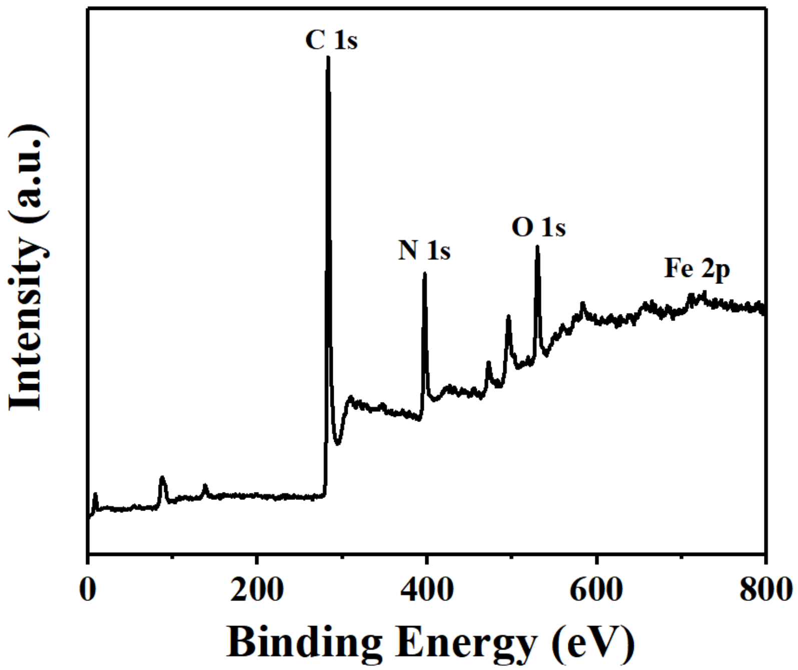

Figure S4. The full spectrum of Fe-N/C-0.05-900

Table S1. The element atomic contents of Fe, C, N and O in Fe-N/C-0.05-900 by XPS measurement

Sample	Element atomic ration (%)			
Fe-N-C-0.05-900	Fe	C	N	O
	1.20	87.76	7.34	3.70

Table S2. The comparison of ORR activity between our work and the references

Catalyst	E_{onset} (mV)	$E_{1/2}$ (mV)	J_{lim} (mA cm^{-2})	Tafel Slope (mV/dec)	Electrolyte	Refs.
**Fe-N/C-0.05-900**	**99**	**88.5**	**4.8**	**52**	**0.1 M KOH**	**In this work**
Fe–N/C-155	100	85	6.0	--	0.1 M KOH	*J. Mater. Chem. A.* **2019**, *7*, 16508-16515. (Ref. 29 in paper)
FeNi0.25-NC	--	86	5.8	--	0.1M NaOH	*Appl. Surf. Sci.* **2021**, *538*, 148017. (Ref. 30 in paper)
C–Fe(OH)$_3$@ZIF-1000	99	88	5.9	--	0.1 M KOH	*ACS Appl. Energy Mater.* **2019**, *2*, 3194-3203. (Ref. 31 in paper)
NC@CoFe,NeCNP	97	86.5	7.99@85mV	61	1.0 M KOH	*Int. J. Hydrog. Energy*. **2021**, *46*, 9341-9350. (Ref. 32 in paper)
Fe–N$_5$/C@G	88	83	5.5	--	0.1 M KOH	*New J. Chem.* **2021**,*45*,13004-13014. (Ref. 1 in ESI)
HP S-doped Fe-N-C	99	91	4.47	56	1.0 M KOH	*Mater. Today Energy* **2021**, *19*, 100624 (Ref. 2 in ESI)
Fe SAs/N-C	101	91	5.8	--	0.1 M KOH	*ACS Catal.* **2019**, *9*, 2158-2163. (Ref. 3 in ESI)
FeNPs@PANI/rGO	106.1	94.2	4.5	64	0.1 M KOH	J.Power Sources. **2020**, *451*, 227733. (Ref. 4 in ESI)

Fe/N/C-48–950–1	99	86	4.8	--	0.1 M KOH	*Electrochim. Acta* **2021**, *366*, 137408. (Ref. 5 in ESI)
Fe-N/C	97.1	84.4	5.68	67	0.1 M KOH	*Int. J. Hydrog. Energy* **2019**, *44*, 27379-27389. (Ref. 6 in ESI)
Fe-N/C	82	72	5.0	55.6	0.1 M KOH	*ACS Catal.* **2017**, *7*, 1655-1663. (Ref. 7 in ESI)

References

[1]. Liu, F.; Yan, N.; Zhu, G.Q.; Liu, Z. G.; Ma, S.Q.; Xiang, G.L.; Wang, S.R.; Liu, X. J.;Wang, W. Fe–N–C single-atom catalysts with an axial structure prepared by a new design and synthesis method for ORR. *New J. Chem.* ***2021***, *45*, 13004-13014.

[2]. Ma, J. J.; Li, J. S.; Wang, R.G.; Yang,Y. Y.; Yin, P. F.; Mao, J.; Ling, T.; Qiao,S. Z.; Hierarchical porous S-doped Fe-N-C electrocatalyst for high-power-density zinc-air battery. *Mater. Today Energy.* **2021**, *19*, 100624.

[3]. Yang, Z. k.; Wang, Y.; Zhu, M.Z.; Li, Z.J.; Chen, W.X.; Wei, W.C.; Yuan, T.W.; Qu, Y.T.; Xu, Q.; Zhao, C. M. Boosting oxygen reduction catalysis with $Fe–N_4$ Sites decorated porous carbons toward fuel cells. *ACS Catal.* **2019**, *9*, 2158-2163.

[4]. Mohammad S. A.; Halima B.;Young-Bae K.; Iron nanoparticles implanted metal-organic-frameworks based Fe–N–C catalysts for high-performance oxygen reduction reaction. *J. Power Sources.* **2020**, *451*, 227733. 5.

[5]. Huang, J.W.; Chen, Y. B.; Yang, J. M.; Zhu, H. B.; Yang, H. Boosting the oxygen reduction performance of MOF-5-derived Fe-N-C electrocatalysts via a dual strategy of cation-exchange and guest-encapsulation. *Electrochim. Acta* .**2021**，*366*，137408.

[6]. Zhang, X. K.; Huang, X.B.; Hu, W. H.; Huang, Y. M. A metal-organic framework-derived Fe-N-C electrocatalyst with highly dispersed Fe-Nx towards oxygen reduction reaction. *Int. J. Hydrog. Energy* **2019**, *44*, 27379-27389.

[7]. Lai, Q. X.; Zheng, L.R.; Liang, Y.Y.; He, J. P.; Zhao, J. X.; Chen, J. H. Metal–organic-framework-derived Fe-N/C electrocatalyst with five coordinated

Fe-Nx sites for advanced oxygen reduction in acid media. *ACS Catal*. **2017**, *7*, 1655-1663.

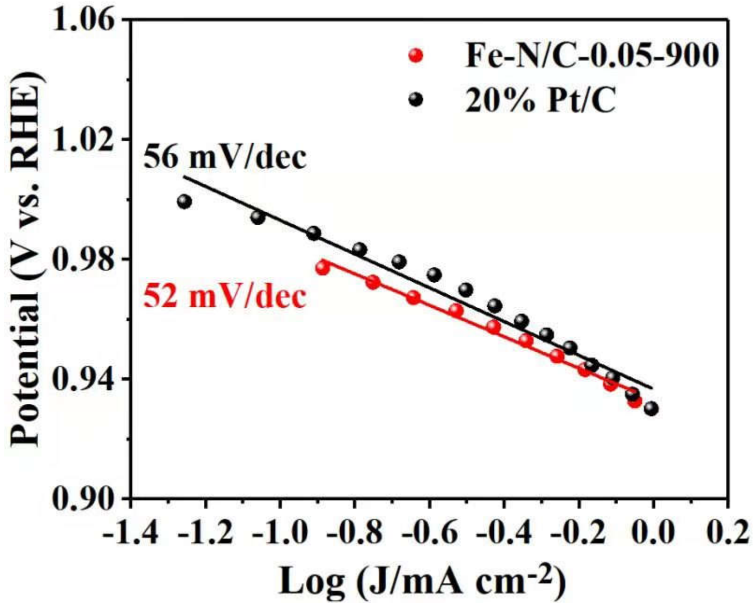

Figure S5. Tafel curves of Fe-N/C-0.05-900 and 20% Pt/C catalysts

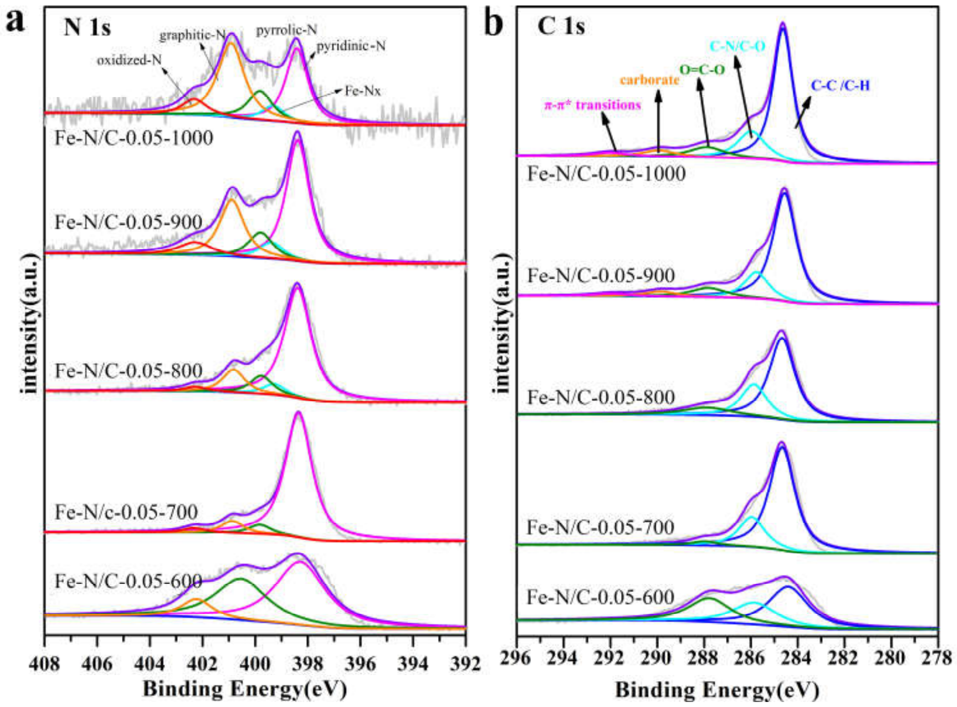

Figure S6. The evolution of XPS N 1s (**a**) and C 1s (**b**) spectra of the Fe-N/C catalysts with increasing heating temperature up to 1000 °C.

Table S3. The N elemental quantification determined by XPS analysis

	Fe-N/C-0.05-600	Fe-N/C-0.05-700	Fe-N/C-0.05-800	Fe-N/C-0.05-900	Fe-N/C-0.05-1000
N at%	16.21	17.71	13.88	7.34	3.69

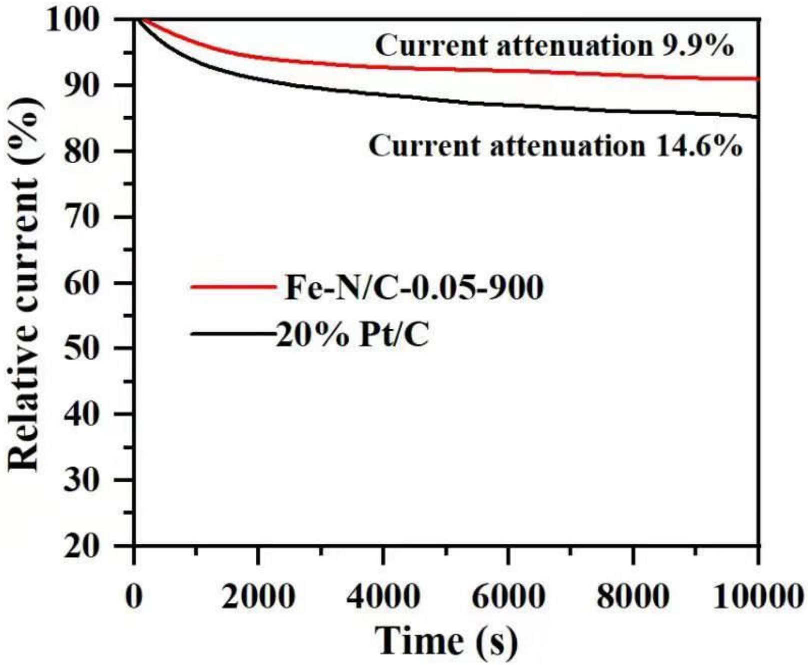

Figure S7. Chronoamperometric curves of Fe-N/C-0.05-900 and 20%Pt/C electrodes in O_2-saturated 0.1 M KOH at a rotating speed of 1600 rpm, the potential was controlled at 0.75 V for 10000 s.
